# Text Mining for Building Biomedical Networks Using Cancer as a Case Study

**DOI:** 10.3390/biom11101430

**Published:** 2021-09-29

**Authors:** Sofia I. R. Conceição, Francisco M. Couto

**Affiliations:** LASIGE, Faculdade de Ciências, Universidade de Lisboa, 1749-016 Lisboa, Portugal; fcouto@di.fc.ul.pt

**Keywords:** cancer, natural language processing, network biology, text mining

## Abstract

In the assembly of biological networks it is important to provide reliable interactions in an effort to have the most possible accurate representation of real-life systems. Commonly, the data used to build a network comes from diverse high-throughput essays, however most of the interaction data is available through scientific literature. This has become a challenge with the notable increase in scientific literature being published, as it is hard for human curators to track all recent discoveries without using efficient tools to help them identify these interactions in an automatic way. This can be surpassed by using text mining approaches which are capable of extracting knowledge from scientific documents. One of the most important tasks in text mining for biological network building is relation extraction, which identifies relations between the entities of interest. Many interaction databases already use text mining systems, and the development of these tools will lead to more reliable networks, as well as the possibility to personalize the networks by selecting the desired relations. This review will focus on different approaches of automatic information extraction from biomedical text that can be used to enhance existing networks or create new ones, such as deep learning state-of-the-art approaches, focusing on cancer disease as a case-study.

## 1. Introduction

Biological networks are a powerful tool to identify different types of information. The type and origin of biological data has a considerable impact on how informative the network is. Some networks are built using heterogeneous data and different aggregation techniques. Networks, such as HumanNet v2 [1], are the result of the combination of multiple data in order to improve network inference methods. This network comes from the incorporation of co-occurrences of protein domains, co-expression of genes across genomic context association, interspecies interactions among evolutionary conserved proteins present in model organisms, inferred functional associations between human genes from protein–protein interactions, and co-citation of human genes across PubMed abstracts [1].

The most common source of information in the last few years has emerged from different high-throughput essays, such as whole genome sequencing, transcriptomics and proteomics [2]. Other sources of biological data can arise from biological process systems as example eye-tracking systems [3] that can help to study complex cognitive process, but this is out of the scope of this review. Nevertheless, most of interaction knowledge is available in the format of scientific literature. In HumanNet v2 network, the co-citation source added a representative amount of information, and this is also true for other networks that use the same type of source, which is the case for STRING network [1,4]. Therefore, it is important to incorporate this source of information into networks, since scientific literature provides newly published information that can be useful to keep these networks updated. One option to extract information from scientific literature is to use human curation. This route provides more reliable information; however, manually extracting from literature is unsustainable [5] due to the large amount of papers necessary to process. Alternatively, this information can be extracted in an automatic way. Nowadays, with the rapid production of scientific literature, the necessity of retrieving information in an automatic way has increased and many text mining tools were developed for this reason [6].

Although text mining relation extraction contributes significantly to information extraction, sometimes this process may result in informational bias due to the focus of some genes and diseases in the research field [1], or have errors associated with the automatic extraction, being these two points of great importance for improvement.

In the context of biological networks, text mining improvement could lead to: (1) an increase in the number of interactions: methods with different sensitivities for biomedical entities would be capable of detecting new interactions, which in turn increase the existing interactions collection; (2) higher confidence on the interactions: as the methods evolve, the more precise they become and less errors on the extractions are present, and by providing evidence from where each interaction is fully described, networks that are more representative of the real system can be achieved; (3) personalized network building: selection of desired interaction pairs and context filters could make it possible to build specific networks, for example, a specific network for a tissue with a given disease.

Fields such as network medicine, which depend on reliable information from networks to make predictions would benefit from advances in text mining approaches to extract relations. This field’s standard approach is to understand human diseases from the perspective of the interactome, so improving text mining methods in order to extract relevant relations would give network medicine a more solid base to construct these interactions and extract useful knowledge.

A disease with a great impact on human health and for that, being one major target of scientific interest, is cancer. Cancer is the denomination of a group of diseases that causes uncontrollable growing of cells and can affect many different organs [7]. According to the World Health Organization, in 2018, more than 9.6 million deaths were caused by cancer, making it the second leading cause of world death [8]. Several reviews that approach the topic about cancer and text mining already exist [9,10,11]. Methods that extract information from unstructured biomedical text with high confidence will provide a reliable source of biological interactions. This will allow to build more stable biological networks that can be explored from various perspectives.

This review will focus on different approaches of automatic information extraction from biomedical text, using cancer disease as a major case study.

## 2. Text Mining

Text mining systems usually employ Natural Language Processing (NLP) techniques to extract knowledge from an unstructured text that was produced by humans [10,11]. The entities of interest are hard to extract, not only because of the heterogeneous text but because the same entity can be written in many different forms, such as diverse synonyms, abbreviations and acronyms. There can also be homonyms, which happens when different entities have the same label [12]. In the biomedical field there is an abundance of variation in terminologies, making text mining tasks more challenging [11] since a term can have different meanings or lead to ambiguities [13]. A practical example of this occurrence can be seen with gene ALK receptor tyrosine kinase (official full name), the official gene symbol is ALK, so it can be referred on text in one form or the other, and besides that, it has two more alternative symbols, CD246 and NBLST3, that can be used to reference it. For cases such as this it is important to normalize all the occurrences on text and link the entity to a knowledge base [11]. Information can be obtained from the full-text or abstracts only. Even though the full-text provides more information, most of the time, only the abstract is used in text mining tasks since it summarizes the most important information, has less information noise, and reduces computational time over-using the full-text [13].

Two main tasks of information extraction systems are: named entity recognition (NER) and relation extraction (RE), which will be explained in the further subsections.

### 2.1. Named Entity Recognition

The goal is to recognize entities present on the text [14]. This is achieved by splitting the text into tokens (words or phrases) and designating them to defined categories. In this task, one of the solutions to circumvent the variation in terminologies is to provide all the alternatives specific to the term in order to normalize it in one entity, using different vocabularies and ontologies [11]. Associated with NER, there can also be Named Entity Linking (NEL) tasks. NEL maps the retrieved entities to a knowledge base. This task mitigates the issue of the synonyms by linking the entity to a unique identifier. In the biomedical context, NER is referred to as a bioNER [15] and is used to recognize entities such as diseases, phenotypes, genes and drugs [11]. These entities can be linked using NEL to the following knowledge bases: Disease Ontology (DO) [16], Human Phenotype Ontology (HPO) [17], National Center for Biotechnology Information (NCBI) [18] and Chemical Entities of Biological Interest (ChEBI) [19], respectively.

Many efforts have been made to create reliable bioNER’s. LSTM-CRF [14] combined deep learning (DL) with distributional word semantics to develop a generic biomedical NER, tested on genes/proteins, chemicals, diseases, cell lines and specie entity types, that is capable of outperforming entity-specific NER tools. CollaboNet [15] also uses a DL approach, which is the combination of single task models (STM) that train on different datasets, being specific for an entity type. These STM are connected to each other and share information between them. This approach was tested on five different datasets of gene/protein, disease and chemicals, achieving an F1 score higher than using a STM for a task. OntheFly^2.0^ [20] is a web application that uses the interactive tool EXTRACT [21], a dictionary-based NER with an high efficient tagger software capable of extracting eight distinct entities (genes/proteins, chemical compounds, organisms, tissues, environments, diseases, phenotypes and Gene Ontology) from diverse document formats and comprises 197 species. Supplementarily the identified terms can be supplied to the STRING database in order to see a network representation of protein-protein and protein-chemical.

Besides dictionary-based bioNER, some state-of-the-art methods use machine learning. One popular method is the bidirectional long short-term memory network conditional random field (BiLSTM-CRF), although, a recent study showed that this method is not good for generalization in another corpus that was not trained on. To surpass this problem the authors, suggest the tool Saber (Sequence Annotator for Biomedical Entities and Relations) [22], which consists of applying different strategies using three modifications: variational dropout, transfer learning and multi-task learning. HunFlain [23], is another recent tool that can identify five biomedical entity types (Cell Lines, Chemicals, Diseases, Genes and Species).

### 2.2. Relation Extraction

Relation extraction task consists of the identification of relations between identified entities in the text of interest. This is one of the most challenging tasks of text mining in biomedical literature due to the complexity that may be found in the sentences, and, in recent years, numerous efforts went into optimizing this task in order to produce results of higher confidence.

Given the example of sentence 1, where the entities of interest are identified:

1. “GENE Guanylate-binding protein 1 (GBP1) promotes PHENOTYPE lymph node metastasis in human DISEASE esophageal squamous cell carcinoma.”

**Figure 1:**
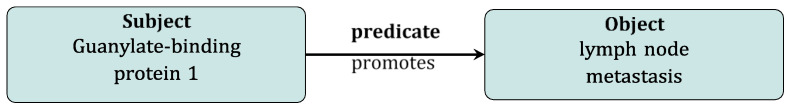
Resource Description Framework example.

Assuming the following recognized entities: “Guanylate-binding protein 1” as a GENE, “lymph node metastasis” as a PHENOTYPE and “esophageal squamous cell carcinoma” as DISEASE. We have the information that the gene Guanylate-binding protein 1 (GBP1) has a role in squamous cell carcinoma; that this gene promotes a lymph node metastasis phenotype and that the lymph node metastasis can be a phenotype of the disease esophageal squamous cell carcinoma. So, it is possible to extract binary relations of GENE-PHENOTYPE, GENE-DISEASE and PHENOTYPE-DISEASE. Binary relationships can be represented by a Resource Description Framework (RDF) triple, which can be interpreted by computers. This type of representation is a subject-predicate-object expression, such as Figure 1:

These RDFs allow the description of data by defining relationships between data objects and permits data integration from diverse sources. Additionally, RDFs can be used to develop a knowledge graph that is a collection of interconnected descriptions of entity-object relations. These knowledge graphs have the capability to present data in context by using ontologies to represent the formal semantics and are deployed using the Linked Data principles. Linked Data principles consist of precise recommendations for exposing, sharing and integrating data, information and knowledge, using semantic web technologies [24], providing a more flexible representation and better knowledge management.

There are multiple approaches to perform relation extraction, one of the simplest assumes that if two entities are mentioned in the same sentence, then they are most likely related (co-occurrence) [25]. The most used methods are supervised learning approaches, which use labeled data to learn and then classify unlabeled data based on learning [26]. In this method, a corpus with the desired entities and their relations is annotated, then it is used to train classifiers that will be able to annotate a test set, which contains novel relations that were not present to the model.

Another method is the pattern-base, which uses manually defined or automatically generated lexico-syntactic patterns to extract relations [27], for instance, using regular expressions to match word patterns that can reflect a relation between two entities. If the targets are drug–gene relations, taking into account the pattern “DRUG-inhibits-GENE”, every sentence where this pattern is identified will be selected.

Another approach is the bootstrap method, which uses a small set of relations denominated as seeds [28], for example drug–gene pair, and finds sentences that include those pairs. Following the context, between and around the entities are generalized in order to map the relation pattern they describe, and these patterns are then used to identify new pairs of the same kind. Using as an example drug-gene seeds in the following dummy sentences 2 and 3:

2. “Drug A activates the Gene A DNA repair response”
3. “Our study suggests that Drug X inhibits the function of Gene A”

The following general patterns are retained: “(…) [Drug] activates the [GENE] (…)” and “(…) [Drug] inhibits the function of [GENE]”. This way it is possible to expand the set of relations without the need of an initial dataset with a large amount of labeled data.

Distant supervision is another method to perform RE. It consists of an automatically generating a large set of labeled data [29]. This is achieved by acquiring sentences with seeds from a large database, then evaluate frequent features in the sentences and train supervised classifiers on the features. In a simpler explanation, it considers that the pairs of entities in any sentence that corresponds to a database entry is likely to describe the relation between the entities, extracting possible labels by pattern and creating a dataset that can be used to train a classifier.

One alternative when there is no available annotated data or knowledge base is the unsupervised RE, in which there is no initial set of relations, and these are extracted from the web, mainly extracting relations expressed with verbs [30]. Although it is a good way to get a large number of relations without specifying them, it is necessary to map the set of strings into some established form.

This variety of approaches to perform relation extraction leads to the development of numerous methods that can be useful in diverse areas, mainly in expanding relations in network biology.

### 2.3. Relation Extraction in the Biomedical Field

Multiple relation extraction approaches have been developed in the biomedical context, demonstrating that useful binary relation extractions such as gene–gene, gene–disease, gene–phenotype, drug–gene, drug–disease or drug–drug can be automatically extracted from literature using text mining techniques.

Although there are a lot of broad-coverage text mining tools, they do not extract biomedical terms as efficiently. An example of this is the Bidirectional Encoder Representations from Transformers for Biomedical TextMining (BioBert), which is a pre-trained model in biomedical corpora [31] based on BERT [32] that is trained on generic text corpora such as Wikipedia. BioBERT was pre-trained on a large scale on PubMed Central full-text articles (PMC) and PubMed abstracts (PubMed), and tested on different datasets of gene–disease protein–chemical for relation extraction, achieving the highest F1 scores on two out of three datasets.

One step to improve the confidence of the extracted relations is to incorporate domain specific ontologies in the relation extraction models. Ontologies provide a common vocabulary with represented shared knowledge [33], and in this scenario it provides domain specific semantics to the models that can help make the connection between semantics and information extraction. Although, this might restrict the modules to be used on a specific domain, some works showed that it improves the classification of the deep learning models [34,35]. Using Long Short-Term Memory (LSTM) and biomedical ontologies, Lamurias et al. [34] incorporated ancestry information from the ontology alongside deep learning, creating the BO-LSTM. BO-LSTM was built to extract drug–drug interactions and showed that incorporating the ontologies improved the classification. Another work that incorporated domain specific ontologies was from Sousa et al. [35], creating the BiOnt model, that improves the previous work from Lamurias et al. [34] by using four types of domain specific ontologies. The used ontologies were Gene Ontology (GO), the Human Phenotype Ontology (HPO), the Human Disease Ontology (DO) and Chemical Entities of Biological Interest (ChEBI) and can be combined in ten distinct relations. Three of these relations were tested on this study: drug-drug, phenotype-gene and chemical-induced disease relations. Results showed that the models performance benefits from the use of ontologies. Other studies suggest the combination of methods to increase performance, such as the study of Zhang et al. [36] that combined recurrent neural networks, which is better at capturing features in more complex sentences and convolutional neural networks, which are better in short sentences. This hybrid approach was tested on five protein-protein and drug-drug interaction corpora and showed a better performance than using the models individually. Another hybrid approach, by Quan et al. [37] uses LSTM with convolutional neural networks to extract protein–protein interactions.

Regardless of whether the majority of the methods focus on binary relations, sometimes more relations can be present. Even more, important relations might not only be in the same sentence, but in connection with the nearest sentences. Provided that some advancements towards the extraction of more than two relations have been made and some in the context of cross-sentences. An example of this is the work of Peng et al. [38] that combines N-ary relation extraction in cross-sentences. This work applies a graph LSTM to identify drug-gene-mutation interactions co-occurring triples using biomedical literature from PubMed Central. This approach showed a better performance than the standard benchmark models used until then. Sentence 4 is an example of the described relations from [38]:

4. “The deletion mutation on exon-19 of **EGFR** gene was present in 16 patients, while the **L858E0** point mutation on exon-21 was noted in 10. All patients were treated with **gefitinib** and showed a partial response.”

A more recent work from [39] also extracts triple relations of drug-gene-mutation, also using the same base approach as [38], but also preserving the word sequence into the document graph. Progressive advances in extracting relations, using more than a sentence and extracting more relations, will provide more knowledge.

### 2.4. Databases with Text Mining Approaches

The assembly of biological networks rely on the information available on several databases (for a comprehensive review about biological databases see [40]). Many databases rely on the advances of text mining tools to extend their interaction collection. One of the most known databases is STRING [4], which gathers a collection of protein-protein interactions for several organisms and relies on predictions using automated text mining. In this database, a statistical approach is used based on co-citation analysis using Online Mendelian Inheritance in Man (OMIM) [41] sources and PubMed abstracts in a large scale.

Another popular database is DisGeNET [42,43,44], containing information of human gene-disease associations and variant-disease associations. This database uses three text mining systems. One of the systems is a NER tool, SETH [45] that normalizes variants of a gene in Single Nucleotide Polymorphism Database (dbSNP) or Universal Protein Resource (UniProt) [46]. The other system is BeFree [47], which is a biomedical text mining tool that performs NER and RE in order to extract the associations between gene-disease and variant-disease. The last one is literature-derived Human Gene-Disease Network (LHGDN) [48] that combines NER and machine learning to extract semantic gene–disease relations.

Open targets [49] also use text mining tools to retrieve target–disease associations. It uses Literature coNcept Knowledgebase (LINK) [50], which extracts relations between genes, diseases, drugs and key concepts by mining titles, abstracts and full text from PubMed literature through the detection of co-occurrences.

Two databases that collaborated in the Biocreative 2010 Challenge III [51], an international text mining challenge, that aimed to use text mining with a different purpose than the ones referred before, are the Biological General Repository for Interaction Datasets (BioGRID) and Molecular INTeraction (MINT). These databases present only curated data and although they do not use text mining to extract relations, they aimed to use it to facilitate the curators work by identifying relevant articles that contain the data of interest [51]. BioGRID archives genetic and protein interactions data from model organisms and humans [52]. MINT is a collection of experimental verified protein–protein interactions [53]. Both databases provided test sets for three tasks of the challenge: gene normalization, which consisted in linking the genes or proteins to a database identifier; article classification that accessed the capability of the systems to retrieve relevant articles only based on the abstracts; and interaction method that compared the manually annotated interactions with the automatic ones [52]. The results showed a positive role of using text mining in aiding the selection of relevant articles for the curators and that overall, these types of collaborations are positive to the biomedical research community [52].

A database that gathers specific information on evidence of disease-gene associations is DISEASES [54]. DISEASES gather information from different sources using text mining. These sources include text mining, knowledge bases and experimental databases. The text mining approaches consist in both NER, with a dictionary-based tagger approach and RE with co-occurrence method.

Table 1 consists of a summary of all the text mining methods cited in this section.

## 3. Cancer and Text Mining

Cancer is a complex disease that has a lot of biomedical literature and clinical reports produced about this topic, and many efforts of the text mining field have focused on extracting knowledge from this continuously increasing literature. There are many different types of cancer with different aspects to consider, and depending on the final scope, different relational information can be extracted, such as: gene-disease—if the gene is associated with the disease; gene-gene—what type of interactions the genes on the disease have; gene-phenotype—what kind of phenotype does that gene influence; gene-tissue—if the gene is expressed in a tissue specific manner; and other types of information.

One of the challenges is the scarcity of structured data that can be read and understood by machines. Some of the initial studies tried to access if NLP could surpass this barrier. A study on breast pathology reports [55] explored the normalization of the reference of entities on a report, as well as for the negation of the entities by defining rules, organizing it in a format that would allow statistical analysis. Although this was an advance, rule-based text processing is still time consuming in terms of the elaboration of the rules.

Jurca and collaborators [13] demonstrated the integration of data mining with network analysis to investigate breast cancer trends. Text mining was used for a large-scale analysis of biomedical abstracts, to generate a hypothesis about breast cancer biomarkers, identifying which genes were more studied across countries and between the years. They used abstracts from PubMed, where they performed terms identification (NER) using BeCAS, which is specialized in biomedical concepts. For RE they used a co-occurrence approach to find gene-gene pairs. Additionally, they explored the relationship of those pairs frequency in the abstracts by using network analysis techniques interpreting the genes as nodes and the co-occurrences within the abstracts as edges. They obtained a connected component with relations reported on ten or more abstracts. This connected network consisted in 1089 nodes and 6815 edges. Ten genes were selected as most important after accessing closeness and betweenness values. These genes were grouped in communities according to their modularity and genes from the same community were manually validated using BioGrid to infer interaction. For one community, four of the five genes had physical interaction and the remaining gene had indirect interaction with the others. In the top ten genes, two were not part of these communities. Investigated separately, the researchers found that one had strong influence in breast cancer and the other, although no experimental data linked it to this cancer, was indirectly connected to others that participate in host signaling pathways possibly involved in cancer.

Another study on breast pathology reports by Yala et al. [56], used a machine learning approach, training the model with manually annotated reports. This study compared their results with Buckley et al. [55] and demonstrated that by using machine learning reduces the manual effort of creating rules without losing accuracy.

An experimental approach of unsupervised learning, combining text mining and pattern mining techniques, was used for relation extraction for breast cancer and affiliated genes in the work of Kawashima et al. [57]. They extracted the related genes from PubMed articles and used them as data in vectors for clustering analysis and joined them with a list of breast cancer related genes. They were compared and clustered in order to extract the candidates. The simple clustering technique, which orders the genes by the lowest to the highest occurrence frequency, obtained a low F1 score (below 0.14).

Exploring biological pathways associated with urothelial cancer, Lin et al. [58] applied a topic model method, which is a probability-based approach to identify topics. This method used Latent Dirichlet Allocation, which is a type of topic modeling, and Lda2vec that is an unsupervised method.

Besides the identification of new genes or pathways, text mining can also help in other biomedical tasks such as registry entries. This was explored by Fabacher et al. [59] that in order to predict if a patient’s data was considered as a prostate adenocarcinoma trained a Support Vector Machine model using pathology reports. The results showed that the method was capable of successfully prefilling the data and could even identify new cases of prostate cancer.

A crucial task in cancer research is to distinguish normal cells from malignant ones. For this there are specified hallmarks of cancer which are characteristics that help to make this distinction (for more information on hallmarks of cancer see [60]). Some works have already tried to classify the hallmarks, such as [61] that uses the DEep Contextualized Attentional Bidirectional LSTM (DECAB-LSTM and [62] that deploys Convolutional Neural Networks.

A study focusing on precision oncology [63], aimed to extract biomarkers from the literature and created CIViC, which is a knowledge base using supervised learning. The developed knowledge base works as a tool to help in the curation of new biomarkers but also as an aid to structure knowledge of clinical relevance by narrowing down the possible biomarkers for the gene and cancer type. This was made using five types of relations: diagnostic, predictive, predisposing, prognostic and associated variant. Each relation was extracted by building a model for each of them. A total of 87,412 biomarkers were extracted with a precision superior of 0.8 (selecting a threshold that had a trade-off of high precision with low recall).

Alawad and collaborators [64] combined a multitask learning technique with convolutional neural networks (MTCNN) in order to extract five cancer characteristics simultaneously (primary site, laterally, behavior, histological type, and histological grade) from cancer pathology reports. The results showed that the two versions of MTCNN that were developed could outperform the conventional machine learning classifiers in the extraction of all five characteristics, with the advantage of extracting them simultaneously instead of one at the time.

A resume of all methods cited in this section is provided in Table 2.

## 4. Discussion and Conclusions

Over the last few years, the field of text mining regarding cancer-related information has improved, and many new approaches have been developed. Approaches have evolved to more sophisticated methods that allow to extract information in a more reliable way. Most of these approaches are shifting to deep learning methods that can extract more than one feature at the time. Methods using complementary approaches, such as ontologies, could also improve information extraction and give more reliable interactions.

The development of text mining approaches is an added value to the biomolecular network field, since this technology can keep up with the most recent literature and is suited to deal with the large volume of new information, thus giving new relations information or revising existing relations to build more updated networks. This could help to narrow the knowledge gap on the interactome level, providing a more solid ground for network method predictions, since the network itself will have more quality. Studies such as the one from Jurca et al. [13] show that it is possible to extract reliable gene-gene relations using text mining that make sense in the network assembly.

The alliance of these fields could lead to more personalized network building, such as building networks that are tissue specific, expanding disease modules information, new protein-protein interactions. Most of the existing biological networks do not offer interaction in a tissue perspective, mixing interactions that can occur in different tissue contexts. This might lead to incorrect predictions. Recent studies in cancer showed that most cancer driver genes are mutated in a tissue dependent manner that is not explained by the gene expression pattern across tissues [65]. This could be an interesting area to pursue in the future, using text mining cancer related information in order to extract enough information for the creation of specific biological networks for each type of cancer.

## Figures and Tables

**Table 1:** Resume of the cited text mining methods.

		Text Mining Task		
Method	Target	NER	RE	Reference
LSTM ^1^-CRFF	Genes/proteins, chemicals, diseases, cell lines and species entity types	X		[14]
CollaboNet	Gene/protein, disease and chemicals	X		[15]
BioBERT	Gene-Disease and Protein-Chemical		X	[31]
BO-LSTM	Drug-Drug		X	[34]
BiOnt	Gene, phenotypes, disease and drugs combinations $\left(\left(\begin{array}{c} 4\\ 2 \end{array}\right)\right)$		X	[35]
RNN ^3^ + CNN ^2^	Protein-Protein and Drug-Drug		X	[36]
LSTM ^1^ + CNN ^2^	Protein-Protein		X	[37]
graph LSTM ^1^	Drug-gene-mutation		X	[38]
graph LSTM ^1^	Drug-gene-mutation		X	[39]
SETH	Gene variant normalization in to dbSNP or UniProt	X		[45]
Befree	Gene-disease and variant-disease	X	X	[47]
LHGDN	Gene-Disease	X	X	[48]
Link	Genes, diseases, drugs and key concepts		X	[50]

^1^ Long Short Term Memory ^2^ Convolutional Neural Network ^3^ Recurrent Neural Network.

**Table 2:** Resume of the cited cancer text mining methods.

Target Cancer	Method	Source	Reference
Breast	Data Mining and network analysis	Biomedical Abstracts	[13]
Breast	Rule Based	Pathology Reports	[55]
Breast	Machine Learning	Pathology Reports	[56]
Breast	Unsupervised Learning, Text mining and Pattern mining	PubMed Articles	[57]
Urothelial cancer	Latent Dirichlet Allocation and Lda2vec	PubMed Abstracts and Titles	[58]
Prostate adenocarcinoma	Machine Learning	Pathology Reports	[59]
Generic	LSTM	PubMed Abstracts	[61]
Generic	CNN ^1^	Biomedical Abstracts	[62]
Generic	Supervised Learning	Full PubMed	[63]
Generic	Multitask CNN ^1^	Pathology Reports	[64]

^1^ Convolutional Neural Network.
